# Neurasthenia at Mengo Hospital, Uganda: A case study in psychiatry and a diagnosis, 1906–50

**DOI:** 10.1080/03086534.2015.1123975

**Published:** 2016-01-11

**Authors:** Yolana Pringle

**Affiliations:** ^a^ Centre for Research in the Arts, Social Sciences and Humanities (CRASSH), University of Cambridge, Cambridge, UK

**Keywords:** Psychiatry, empire, Uganda, neurasthenia, mission medicine, detribalisation

## Abstract

This article uses a case-study approach to examine the complex and contradictory nature of diagnoses like neurasthenia in colonial Africa. Drawing on the case notes of European and African patients diagnosed with neurasthenia at the Church Missionary Society's Mengo Hospital, Uganda, it argues that in practice, and outside the colonial asylum in particular, ideas about race and mental illness were more nuanced than histories of psychiatry and empire might imply. At Mengo, the tales of pain and suffering recorded by the doctors remind us that there is more to the history of neurasthenia than colonial anxieties and socio-political control. This was a diagnosis that was negotiated in hospital examination rooms as much as in medical journals. Significantly, it was also a diagnosis that was not always reserved exclusively for white colonisers—at Mengo Hospital from the early 1900s neurasthenia was diagnosed in African patients too. It became part of a wider discussion about detribalisation, in which a person's social environment was as important as race.

## Introduction

One of the key features of psychiatry in colonial Africa was the way in which understandings of mental illness intersected with assumptions about racial difference. The African and the European ‘mind’ were considered to be biologically and culturally distinct, with mental illness, by extension, differing in both cause and appearance.^1^ Research on ‘the African mind’, as it became known, encompassed studies of African intellect, personality traits and psychopathology, and maintained that a number of mental illnesses frequently seen in America and Europe, such as depression and the neuroses, were completely absent in Africa. More overt forms of ‘mental derangement’, by contrast, were increasingly common, as ‘detribalisation’—something that encompassed such diverse aspects as ‘Christianization, secular education, working relationships with non-African employers, relationships with Government officials and with shop-keepers … life in townships, and the introduction of syphilis and alcoholic spirits and other drugs’—took hold.^2^

Neurasthenia, a condition of ‘nervous exhaustion’, was one such illness that took on extended racial connotations when it was transported from America and Europe into the colonial context.^3^ First described in 1869 by American neurologist George M. Beard, neurasthenia covered a wide variety of physical and mental complaints, including headaches, insomnia, listlessness, irritability, anxiety, abdominal pain and profound fatigue.^4^ It signalled a disease of the nervous system, but was a condition that on examination had no identifiable physical cause.^5^ In the colonial context, ‘neurasthenia in the tropics’ or ‘tropical neurasthenia’, differed little from its metropolitan variant, with one key exception: in the medical literature at least, it became associated almost exclusively with the mental and physical breakdown of white colonisers. While the causes of tropical neurasthenia were the subject of intense discussion, there was little doubt among medical officers and administrators that neurasthenia represented one of the major causes of being invalided home from the tropics. Feeding into debates about acclimatisation, neurasthenia became associated with the strains of living in ‘unhealthy’ environments among ‘uncivilised’ tribes. It reinforced ideas of racial difference already presumed by colonial rule, positing white ‘nerves’ against a more dangerous and unpredictable black ‘madness’.

This article uses a case-study approach to examine the complex and contradictory nature of diagnoses like neurasthenia in colonial Africa. Drawing on the case notes of European and African patients diagnosed with neurasthenia at the Church Missionary Society's Mengo Hospital, Uganda, it argues that in practice, and outside the colonial asylum in particular, ideas about race and mental illness were more nuanced than histories of psychiatry and empire might imply. Neurasthenia provides the ideal subject for a case study of this kind because, as a medical construct, it has been shown to be revealing of wider colonial anxieties.^6^ Using medical journals, memoirs and administrative records, historians have linked the diagnosis with ideas about acclimatisation, masculinity and white deviancy, arguing that neurasthenia not only provided a socially acceptable explanation for European and American dysfunction, but a means of regulating the behaviour of colonisers in the tropics. Such was the use of the diagnosis for ‘policing the colonizers’, Anna Greenwood has argued, that medical officers in British East Africa continued to publish on neurasthenia and its relationship to acclimatisation until well into the 1930s, despite calls from medical authorities in Europe to abandon the diagnosis.^7^

This article introduces patient case notes from Mengo Hospital into the historiography of tropical neurasthenia and empire. At Mengo, the tales of pain and suffering recorded by the doctors remind us that there is more to the history of tropical neurasthenia than colonial anxieties and socio-political control. This was a diagnosis that was negotiated in hospital examination rooms as much as in medical journals. Significantly, it was also a diagnosis that was not always reserved exclusively for white colonisers—at Mengo Hospital from the early 1900s neurasthenia was diagnosed in African patients too. It became part of a wider discussion about detribalisation, in which a person's social environment was as important as race. In this way, the breakdown of Africans whose contact with civilisation had supposedly opened them up to mental illness could be compared with the breakdown of Europeans who had been physically and psychologically unprepared for life in Africa.

Mengo Hospital was founded by Albert Ruskin Cook of the Church Missionary Society (CMS) in 1897. Its opening marked the start of increasing CMS preoccupation with medical work in central and eastern Africa, as well as the strong medical missionary presence that came to characterise medicine in early colonial Uganda.^8^ While some patients were referred by missionaries working across Uganda, most came from the locality (Mengo/Kampala), being brought in by friends, relatives and employers. Mengo Hospital had a particularly close relationship with the local Buganda government, whose kabaka (king) and katikiro (prime minister) were both attended to by Cook. In addition to supporting the hospital through donations of labour, land and building materials, the Buganda government actively encouraged the Ganda to visit the hospital for all types of illness and disease, including mental illness.^9^

At a time when colonial government provision for mental illness was limited to the confinement of a small number of psychotic patients, general hospitals such as Mengo provided limited care to those seeking relief from minor mental illness.^10^ These hospitals were not institutions of confinement; rather, as general hospitals, they attempted treatment on a short-term basis, and allowed friends and relatives to retain control over their patients. They were not always enthusiastic about providing care for those in psychological distress, and those that were difficult to manage were frequently turned away or discharged after only a few days. This type of hospital setting, catering for a wide range of physical and mental ills, made it ideal for those who found themselves suffering from one or more of the symptoms of neurasthenia, and there is evidence to suggest that they appeared at hospitals elsewhere in Uganda, too.^11^

Over the period 1897 to 1950, the case notes and admissions registers of Mengo Hospital, located at the Albert Cook Library, Makerere University, and in the Records Office of Mengo Hospital, Kampala, indicate that at least 558 patients, of all races, were admitted and diagnosed with some form of mental ill-health, including hysteria, neurasthenia, dementia, schizophrenia and psychoneurosis. While the level of detail in the case notes tends to vary by the race of the patient, as well as the interest, knowledge and time of the doctors, the collection, which forms an almost unbroken record of hospital medicine from 1897 to the present day, is an exceptional source for medical historians in Africa, and certainly for those in Uganda.^12^ An analysis of these case notes provides opportunities not only to examine diagnoses across racial groupings, but to explore the ways in which ideas about mental illness and race were understood and deployed in a hospital setting. This was a context in which doctors made practical decisions about what constituted neurasthenia—decisions that involved on-going negotiations between doctor and patient. It is therefore perhaps unsurprising that understandings of race were much more nuanced in practice than in the tidy, ordered world of the published medical literature.

## Neurasthenia and race in the medical literature

In exploring the relationship between race and tropical neurasthenia, it should be remembered that racial understandings of nervousness were not unique to the colonial context.^13^ As neurasthenia became associated with concepts of degeneration in America from the late 1890s, the appearance of neurasthenia in black Americans was increasingly noted.^14^ Neurasthenia, it was said, was particularly common in ‘half-breeds’, being brought on by the ‘struggle of the best specimens of an inferior race to attain the plane of a superior [one]’.^15^ These sentiments were repeated and reinforced later in the colonial context as attention shifted in the 1920s from an almost exclusive focus on the nervous strains of white colonisers to the psychological stability of indigenous peoples living under colonial rule.^16^ Before this, comments on the occurrence of neurasthenia in ‘natives’—by which authors meant those who were neither white nor American or European—appeared only as after-thoughts in articles focusing on the health of white colonisers. Preoccupied with the dangers of climate, the cost of being invalided out and the long-term sustainability of colonialism, the medical literature debated and refined understandings of neurasthenia in Americans and Europeans alone.

As early as 1906, W. W. King, a Passed Assistant Surgeon in Washington DC, claimed that, while neurasthenia did occur in Puerto Ricans, it was far more common in those coming from ‘colder countries’. As such, it was ‘a very interesting question to American physicians, in view of the close relations recently established between the United States and Porto Rico [*sic*], Cuba, [and] the Philippines’.^17^ While King continued to discuss the ways in which lifestyle, climate and hygiene might bring on the condition in white colonisers, he had acknowledged the occurrence of neurasthenia in Puerto Ricans. This, he qualified, was usually ‘encountered associated with hysteria—hystero-neurasthenia', an ill-defined medical construct that was avoided by most medical authorities; King did not add much by using the term hystero-neurasthenia. But his emphasis on how neurasthenia presented differently in Puerto Ricans is suggestive considering the importance medical contemporaries placed on the role of the nervous system in the development of functional disease.^18^ If Americans and Puerto Ricans were biologically different, mental illness, by extension, would also differ in cause and appearance.

Such was the belief in biological difference between the races, particularly before the 1920s, that a number of medical commentators questioned whether natives could even suffer from neurasthenia. Drawing on the original conceptions of the condition as formulated in America and Europe, medical officers stressed how neurasthenia was a disease of modernity and ‘civilisation’—the burden of ‘nerves’ was one shared only by the superior, colonising races.^19^ Charles F. Harford, of the CMS, reinforced this point in a discussion on the potential role of electricity in causing neurasthenia among Europeans in India. He asserted that he had heard on good opinion that the Japanese did not suffer from ‘Japan head’, a term used by the CMS to describe the nervous troubles reported by their missionaries in the far East. For Harford, this was a sign that ‘electrical influences’ in the tropics affected European but not Japanese nervous systems.^20^

Mirroring a more general shift in interest towards the health of the colonised, the 1920s saw an increase in comments about neurasthenia in different races. These comments also tended to focus less on whether natives could suffer from the condition and more on the reasons why it might occur. In one of the first sustained discussions on neurasthenia in ‘coloured individuals’, recorded at a meeting of the Royal Society of Tropical Medicine in 1927, H. M. Hanschell noted that he knew of cases of West Indians becoming neurasthenic in London. He recalled how:

[w]hen these cases came to the Seamen's Hospital in the Albert Dock they invariably complained of physical illness—which thorough examination failed to confirm. On obtaining their confidence, an essential, though often a long business, one found anxiety and mental conflict; and back [*sic*] of it all a desire to return to the West Indies’.^21^

Following Hanschell, Hugh S. Stannus confirmed that he had also seen neurasthenic symptoms in Africans. Using a language reminiscent of debates in Britain on the degeneration of the working classes, Stannus added that:

I, too, have seen neurasthenic symptoms in such people in this country, but also in natives in their own countries—not while living their normal lives in their villages, but when partly educated and subjected to conditions to which they were not adapted—just as one sees neurasthenia common to-day among a partly educated class whose immediate forbears [*sic*] were uneducated.^22^

These ideas had roots in race- and class-based understandings of neurasthenia as it had developed in its original American and European contexts. But they were also reinforced by wider shifts in thinking about ‘culture contact’ and ‘detribalisation’. From the 1930s, scientific communities in Africa started to discuss how contact with western civilisation could ‘detribalise’ the African and trigger a particularly ‘European’ type of insanity, characterised by delusions of power and control.^23^ Within East Africa, these ideas achieved coherence in the pages of the *East African Medical Journal* (*EAMJ*), where psychiatrists and psychologists set out their theories on African thought processes, personality types and behaviours. While there was some disagreement about the role of race and culture in either predisposing Africans to mental illness or providing a form of ‘psychotic immunity’, all of the research was guided by the assumption that western civilisation was dangerous for ‘primitive minds’.^24^ This body of literature would later come to be known as the East African School of psychiatry and psychology, achieving infamy in the 1950s for the vociferous arguments of J. C. Carothers about ‘the African mind’.

While the psychiatric literature on detribalisation focused on the more unpredictable and disturbing forms of mental illness, such as mass hysteria, it was rarely linked to conditions that in their original European contexts were considered to affect only the ‘civilised’ races.^25^ Discussion of neurasthenia in natives was by contrast limited primarily to non-specialists, something that goes some way in explaining why they came into contact with people exhibiting neurasthenic symptoms at all. Certainly, neurasthenia was a condition that was not found in the colonial asylum, just as it was not found in asylums in Europe or America. It was the general clinic or urban workplace where these ‘neurasthenics’ were encountered—places where they were not disruptive and posed no danger to family or community. The CMS Mengo Hospital, to which this article now turns, was one such setting.

## Neurasthenia at Mengo Hospital

If there was some question in the medical literature as to whether Africans could suffer from neurasthenia, particularly before the 1920s, this was certainly not the case at Mengo Hospital. Between 1906 and 1950, 57 Africans were diagnosed with the condition and treated as in-patients. The first recorded case occurred in 1906, a time when the medical literature still focused on the appearance of the condition in white colonisers. Over the next 14 years, a further five Africans—male and female—were diagnosed with the condition. While these numbers were small, they were comparable both to the number of Europeans diagnosed with the condition and to the figures released by the colonial government showing the number of European officers invalided home each year due to neurasthenia. Over the years 1911–15, for example, two Africans and ten Europeans were diagnosed with neurasthenia at Mengo Hospital, while one European official was invalided from Uganda.^26^

Of course, the total number of Europeans living in Uganda during this time was significantly smaller than the number of Africans. The estimated population for 1918, for example, showed 570 Europeans (313 males and 257 females) and 3,357,080 Africans.^27^ Moreover, neurasthenia cases made up only a small fraction of the patients admitted to Mengo Hospital—in 1908, when Mengo Hospital had 120 beds and had seen 1,408 in-patients over the year, there was only one diagnosis of neurasthenia (in an African male).^28^ Nevertheless, the presence of the diagnosis in the patient case notes indicates that from early on in Mengo Hospital's history, contrary to much of the medical literature on the subject, the doctors not only believed that Africans could suffer from neurasthenia, but that they could recognise it and treat it in a mission hospital.

The number of African cases started to rise after 1920, mirroring the acknowledgement in the medical literature that the condition could occur in any race. The number of cases at Mengo Hospital peaked in 1931–40, when at least 35 neurasthenic patients were admitted. The phenomenon was short lived, however—the diagnosis waned during the 1940s, dying out completely after 1950.

Diagnoses of neurasthenia in the European patients followed a quite different trajectory, with the highest number of cases occurring between 1911 and 1920. While there are no surviving case notes for Europeans diagnosed with neurasthenia before 1911, it is clear from other documents that at least three CMS missionaries were invalided home from Uganda with neurasthenia in the 1900s. All of these missionaries would have been required to see brothers Albert or Jack Cook for a medical certificate before their departure.^29^ If they had been formally admitted to the hospital, their records have probably been destroyed or misplaced—before the opening of a private European ward in 1910, and the subsequent acquisition of European admission registers and casebooks, notes on the Europeans treated at Mengo Hospital were frequently left loose. The total number of European patients admitted for any condition also rose steeply with the opening of the European ward, from 37 in 1910, to 78 in 1911, to 110 in 1912.^30^

Significantly, for a condition that in the early medical literature was associated almost exclusively with the breakdown of white colonisers, neurasthenia was the most common diagnosis of mental ill-health for both African and European patients in the 1930s. Between 1931 and 1940, of the 101 African patients admitted with some form of mental health problem, 35 cases of neurasthenia were diagnosed.^31^ During the same period, of the 12 European patients admitted suffering from mental ill-health, 10 cases of neurasthenia were diagnosed. As such, the prevalence of the diagnosis raises questions about understandings of neurasthenia at Mengo, particularly in the 1930s: was neurasthenia considered to present differently in European and African patients? Was there a shift from somatic to psychological understandings of the condition? And did the doctors at Mengo look more to British or to ‘colonial’ understandings of neurasthenia?

It should be noted here that in all cases at Mengo the diagnosis was that of ‘neurasthenia’, rather than ‘tropical neurasthenia’. This is not surprising considering that the terms ‘neurasthenia’ and ‘tropical neurasthenia’ were used interchangeably in the medical literature. As Greenwood has pointed out, the differences between neurasthenia and its tropical variant were mostly ‘in emphasis rather than kind’, with particular weight placed on the sun as a causative factor.^32^ That the doctors at Mengo Hospital used the term ‘neurasthenia’ could thus have been an admission that the ‘tropical’ in tropical neurasthenia tended to refer more to a sense of locality than ‘to characterize any special type of neurasthenia’.^33^

Neurasthenia had always been a ‘protean’ condition, and, as Freudian theories on the soma and psyche rose in prominence in Europe, environment was increasingly discounted as a predisposing factor for nervous breakdown.^34^ Anderson has argued that with these shifts the ‘tropical’ in tropical neurasthenia lost its relevance: ‘Not simply a potentially avoidable physiological failing of the white race in an alien land, neurasthenia became … a sign of wilful individual disaffection with modern life, evidence of deep-seated mental conflict, of the family drama.'^35^ In contrast, Greenwood has demonstrated how in British East Africa, ideas about neurasthenia as related to topography persisted in published medical literature until well into the 1930s.^36^ The case notes at Mengo Hospital suggest that in general medical practice in East Africa, too, neurasthenia persisted as a label for mental and physical breakdown that was very much linked to a person's social and physical environment. How the doctors and patients understood and applied the condition at Mengo Hospital is the subject of the following two sections.

### European patients

The process of examination for the European patients at Mengo showed a strong emphasis on the search for somatic signs of illness. As it was in George M. Beard's original outline of the condition, the diagnosis of neurasthenia was one reached as much by a process of exclusion as through the imposition of pre-conceived notions and ideas.^37^ The most common symptom was pain that on examination could not be linked to any organic cause. Jeanne B.’s pains had prompted her to ask Albert Cook in 1930 ‘whether she had appendicitis’, yet upon examination this and a range of other physical explanations for her pain were ruled out.^38^ Edith D. had been suffering from headaches, giddiness and nausea for some time, and in the week before her admission had also developed shooting pains in her leg. Her examination involved checks on her cardiovascular system, throat, abdomen, teeth and urine, which turned up nothing abnormal except for excessive urination.^39^ In a similar case, Dorothy F. was also put through a thorough physical examination after she stated that she had been troubled by irregular pains ever since she had ‘sat on the luggage carrier of a motorcycle’ in England two years before.^40^

In the case notes, the search for physical signs was interspersed with notes on the patient's place of residence and time spent in East Africa, suggesting that climate was considered to play a role in predisposing an individual to the condition. Guy R. had suffered from ‘restlessness, shakiness + severe pain in the back of the neck’ for three weeks when he arrived at Mengo Hospital. He had a long history of travel across East Africa, which by the time of his admission in 1933 stretched over 10 years. Guy had recently spent time in the sun, and, while he reassured his doctor that he had worn a sun helmet, it was noted that the pain ‘may have been due to the effect of exposure to the sun’.^41^ In another case in 1935, the doctors at Mengo Hospital felt it noteworthy that Sarah O., who had experienced weight loss and periodic pain for over a year, was always away on safari.^42^

As highlighted earlier in this article, Greenwood has argued that colonial medical officers in British East Africa ‘noticeably lagged behind the changing psychiatric fashions of the 1920s and 1930s, frequently still publishing on acclimatization and theories of neurasthenic breakdown relating to place’, something she in part attributes to a preference for older doctors.^43^ The doctors at Mengo, too, continued to link climate with the mental and physical states of their patients until well into the 1930s. Greenwood's argument, however, draws too fine a line between the climatic and psychological aspects of the condition—the divide between neurological and psychological interpretations of illness was rarely clear-cut, and general practitioners in Britain and America continued to use neurasthenia as a convenient label for mental and physical breakdown. As Mathew Thomson has noted for Britain, ‘With its amorphous character, neurasthenia proved adaptable to the changing climate of opinion … acknowledging that mental worry might precipitate bodily ills left such ills no less real for this; and it left the possibility that the same was true in reverse’.^44^

While the emphasis on the soma and climate persisted throughout the period at Mengo, it was rarely divorced from the more psychological aspects of the condition, particularly from the 1920s. Vera H., a female patient diagnosed with neurasthenia in 1932 had been in Uganda for two years when she presented herself at the hospital. As with the other patients, Vera was examined for physical signs of illness, and her length of time spent in Uganda noted. At the same time, her problem was presented as in part psychological, in so far as her doctor, Robert Stones, noted that she ‘[d]oes not like [the] country [and] wishes to return to England’.^45^ For E. L., too, it is unclear whether the doctor placed more weight on his travels around East Africa without quinine or an ‘[e]motional shock’, which had ‘brought back trouble with [right] ear … pain, giddiness, nausea and nervousness’.^46^

More problematic is the case of Marie G., a Belgian Protestant missionary who was in certain respects the archetypal tropical neurasthenia patient: she had been ‘rescued’ from her station in Rwanda by CMS missionaries, having been alone at the station for five years.^47^ Her isolation may have played a key role in her diagnosis, and that she was sent back to Belgium highlights a related assumption about tropical neurasthenia—that the symptoms would disappear if the patient changed their location or returned to Europe.^48^

The variety found in the case notes suggests that, in practice, understandings of neurasthenia could not be divided easily into the climatic and the psychological. Not only was this distinction frequently blurred in the medical literature, but the doctors also brought their own understandings and priorities to the examination room. Judgements about the ‘nerves’ and character of missionaries played an important role in missionary recruitment and in the annual medical examinations of missionaries in the field.^49^ Moreover, in a medical certificate produced for the Old East African Trading Company in 1929, Albert Cook described Richard M. as suffering from ‘morbid depression, kleptomania, insomnia and a dread of impending mental trouble’. These symptoms, Cook stated, suggested that a diagnosis of neurasthenia was suitable, and as such Richard was ‘in my opinion quite unfit for continued residence in the Tropics’.^50^

Such cases appear to have been exceptional, however. There are no references in the case notes to behaviour that might have been in need of ‘policing’, such as the tendency to drink, steal or engage in inappropriate relationships. Instead, the patients nearly always arrived at the hospital because they themselves felt they were ill and were discharged back to their homes or workplaces after brief admission periods. For the doctors, neurasthenia was not simply an intellectual construct, serving a socio-political role. Working under the pressure of needing to complete as many consultations as possible and with limited options for referral, these doctors also had to contend with the expectations and assumptions of their patients.

One such assumption was that Mengo was among the best for Europeans in East Africa. This was partly due to the facilities available at the European-only Annie Walker Ward, but also due to the reputation of Albert Cook.^51^ Hundreds of letters from Europeans seeking advice, diagnoses and medication survive in the archives of Mengo Hospital. They bear testament both to Cook's notoriety and to the personal suffering of those seeking relief from mental and physical dysfunction. In one letter from a European living in Kisumu, Kenya, the author complained of a wide range of symptoms, including ‘ringing in the ear’, ‘lassitude or lack of energy’, ‘loss of appetite’, ‘inclination for vomiting’ and ‘night emissions’. Drawing attention to his long search for a diagnosis that he could accept, the author reminded Cook how:

I have undergone the treatment of four physicians each of them has treated my case differently, i.e. one said I was suffering from hearth-weakness [*sic*] + nervousness, the other dyspepsia, the third chest disease, the fourth piles, etc, and this has been done on me since October last, now, as a desperate man, I have come in search of you, the only famous + celebrated Doctor in the country for first resource and decision, and to whom I have thought to be the only man who will put me right.^52^

The case notes indicate how some patients had endured years of mental and physical problems prior to their admission to Mengo. Florence C. had suffered from pain in her pelvis, back and upper thighs for five years by the time she presented herself at the hospital in 1912. She had already consulted a number of other doctors, including Dr Johnstone of Penrith, who had ‘removed a polypus [and] operated for haemorrhoids’, and Dr. Playfair, who ‘per contra said it was “all imagination”’, and had prescribed his famous ‘rest-cure’.^53^ Similarly, Alice S. had a ‘neurasthenic history’ of ‘vague pains’ and ‘fears’ that stretched back years. On arrival at Mengo in 1935 she told the admitting doctor, A. T. Schofield, that she had already seen other physicians and been subject to numerous X-rays, all supposedly to little effect.^54^ Whether or not the recounting of these histories of suffering played a role in the diagnosis—they probably did—they show something of the experience of these patients in their search for relief. They were not only looking for treatments that would relieve their suffering, but a diagnosis that would validate their complaints.^55^

The implications of a diagnosis of neurasthenia varied. Although neurasthenia could indicate violent or deviant tendencies, it was primarily a socially acceptable diagnosis that did not carry the stigma of ‘real’ mental illness.^56^ A short period of rest, ideally at altitude, was believed to help relieve the condition, and the doctors at Mengo were well practised in providing advice on suitable locations. For some patients, however, the diagnosis provided a justification for a return to Europe, perhaps indefinitely and at the expense of the employer. This was the case with Edith C., wife of an officer in the King's African Rifles, who wrote to Cook in 1914 asking for help. She had suffered from a bad throat, piles, ‘pains and aches all over' and generally ‘felt very seedy’, for which she had decided on a trip to Kijabe, ‘but the elevation did not suit either of us’. Through self-medicating, she had also become reliant on Sanatogen, iron, quinine and strychnine, a highly toxic central nervous system stimulant. A few months later, Edith was admitted to Mengo and diagnosed with neurasthenia, before being invalided back to the United Kingdom on a medical certificate.^57^

As in Britain and elsewhere in the colonies, neurasthenia provided an explanation for a vast range of ailments that ‘successfully satisfied the needs of doctors and patients within the medical marketplace’.^58^ At Mengo Hospital, the idea that ‘nerves’ could cause mental and physical breakdown would have been familiar to most of the European patients, particularly as popular literature and advice on ‘nerves’ and environment continued to circulate until well into the 1930s.^59^ As late as 1946, the ‘Health Instructions for Missionaries Overseas’ stressed the dangers of climate, even if alongside more psychological strains. It reminded readers how ‘[a]lteration of environment and strains in one's work and personal relationships are very frequent causes of nervous exhaustion. More missionaries are invalided under this heading than from more definite diseases.'^60^

At Mengo, not only did the patients recount years of nervous troubles, but drew on this language of nerves to describe how they were feeling. Mrs H. told her admitting doctor that she had been ‘feeling run down [and] “nervy” recently’, although it is likely that her longer history of being ‘mentally afflicted after [the] birth of [her] child in 1926' was also important.^61^ Likewise, W. A., weakened by influenza and fever, complained of feeling ‘all-to-pieces [and] physically run down’.^62^ In the context of the hospital examination room, it is unsurprising that the doctors at Mengo Hospital held on to a diagnosis that would be accepted and understood by their patients. Even if they were aware that neurasthenia had fallen out of fashion as a diagnosis, it could still serve a function.

### African patients

While the Europeans may have expected ‘nerves’ to be the cause of their complaints, this was not necessarily the case for the African patients. Remarks on the mental state of the patient were always written from the perspective of the admitting doctor—a patient would ‘look nervous’ or be ‘of a nervous disposition’. While this could indicate that the language of ‘nerves’ was one shared by Europeans alone, it also demonstrates some of the cultural difficulties faced by both doctor and patient in the examination room. As John Orley noted in his study of mental illness among the Ganda, ‘Patients often present a series of complaints about aches and pains which in many cases reflects the difficulty that the patient has in expressing himself (and possibly his desire to express his illness in what he thinks are terms acceptable to western medicine)’.^63^ G. Allen German similarly highlighted the preference for the language of the body in his study of depression in 1972. He noted that it was only those patients who had been most exposed to western culture through the school system who ‘used terms like “depression” spontaneously’, and who ‘admitted to being sad or unhappy when questioned directly’.^64^

Certainly, while the European patients talked of feeling tired, overworked or run down, the African patients drew on physical language alone to complain of vague pains, insomnia, heart palpitations and giddiness.^65^ Yaeri N. had suffered from a number of pains before she presented herself at Mengo Hospital in 1918. The admitting doctor noted that ‘[f]or sometime [she] has had considerable vaginal discharge [and] burning pain on micturition [urination]’, as well as pain in her abdomen, head and temples.^66^ On physical examination, however, no organic cause could be found for her complaints. Likewise, the doctor could find nothing physically wrong with Mikaeri L., who in 1908 was reported to have been ‘taken ill 10 days ago with dryness of throat [being] unable to swallow his saliva. 2 days later he suffered from headache and giddiness [and] fever pains all over [his] body. He has had these symptoms everyday until now.'^67^

The emphasis on the search for somatic signs of illness highlights the similarities rather than the differences in the examination of African and European patients. Where there was a difference was in the relative occurrence of the condition before the 1930s: while neurasthenia was the most common diagnosis of mental ill-health in Europeans during the 1910s and 1920s, the African patients were far more likely to be diagnosed with hysteria. These cases of hysteria were usually clearly distinguished from neurasthenia—the doctors emphasised the presence of ‘hysterical outbursts’, the feeling of a ball in the throat, spontaneous movements, jerks and fits. A few cases, however, were remarkably similar. Mai N. had been suffering from pain in her abdomen for two weeks when she presented herself at Mengo Hospital in 1908. A physical examination conducted by Cook found that her abdomen was slightly distended, but otherwise no abnormal signs of illness could be found.^68^

There was no gendered aspect to either the hysteria or neurasthenia diagnosis at Mengo before the 1930s. Yet comments on the patient's occupation, education and schooling suggest that the doctors, as with their European patients, might have brought their own expectations to the examination room. The first recorded case of neurasthenia was in Daudi M., a male Ganda chief who arrived at the hospital in 1906 and complained that he had been feeling unwell for many months. According to Cook, Daudi looked depressed, and, following a physical examination, Cook noted that he was not anaemic, his tongue was ‘slightly coated' and he contracted ‘his abdominal muscles in a curious way’. The only clue as to why Daudi was diagnosed with neurasthenia comes in his position as a chief. Staying in the chiefs' ward would have elicited higher patient fees, perhaps making Cook more amenable to his admission.^69^ But it could also have indicated the ways in which neurasthenia was associated with class. Just as neurasthenia was said to be an affliction of the ‘superior’ races, it was also one that was believed to affect those of higher mental ability within a particular race.^70^ As such, the diagnosis of neurasthenia in Daudi could be seen as a recognition that Cook felt his position as a chief set him apart from other Africans at the hospital.

There are no clues as to why the other eleven cases were diagnosed with neurasthenia before the 1930s. In these cases the doctors made only brief notes on the symptoms presented by the patients and the tests performed during the examinations. If there was any aspect of the diagnosis that was based on class, race or gender, it was not made explicit in the case notes. In the 1930s, however, the number of cases of neurasthenia overtook those of hysteria, becoming the most common diagnosis of diagnosis of mental ill-health for African patients. The case notes also become fuller, including occasional notes on the mental ability and educational progress of the male patients.^71^ These comments do not dominate the case notes, and at times it is difficult to distinguish between notes taken for general information and notes that the doctors felt indicated a form of pathology, but their presence suggests that neurasthenia may have been diagnosed more often in those Africans who were receiving an education.

Erokano D. had been ill with fever for three weeks before his symptoms worsened to the extent that he was taken to Mengo. The day before his admission he had ‘started to vomit … did not know what he was doing’, ‘talked nonsense' and ‘acted strangely’. His doctor listed previous illnesses, including fever and cough, but also pointed to the strains of school life. He had reportedly been ‘working hard at school’, and, while ‘[r]ather dull mentally at present’, looked ‘intelligent [and] answers questions’. Significantly, he was ordered to ‘rest at home’ and ‘[n]ot to go to school’ for at least two weeks, when he should return to the hospital for further inspection.^72^ In a similar case, Charles N. was 16 years old when he was admitted to Mengo. He had come from King's College Budo, a CMS school that was widely noted as Uganda's top training ground for future leaders.^73^ Charles had suffered from a fit and had vomited that morning, having been unwell for a long time. Neurasthenia was not the first thing the admitting doctor, Schofield, had considered. Probably because of the fit, Schofield noted down that there was no previous history of epilepsy in the patient or in his family and, after a physical examination, ‘[n]o evidence of epilepsy’ was seen. Further examination found nothing abnormal in his stool, blood or urine, and after a week in the hospital he was sent for an examination with the head doctor, R. Y. Stones, before returning to Budo.^74^ A student at Mukono College, a religious training school, had also been sent to Mengo after he had suffered from ‘pain in [his] back’ for two weeks. Having been examined at a government dispensary and at Mengo on a previous visit, he was no better. The admitting doctor, Stones, noted that he still complained of ‘increasing pain in back [and] central renal region’ and that ‘his urine has been red for 1 month’. Following his admission, the patient continued to complain of ‘pain in chest’, for which none of the doctors could find any organic cause.^75^

These references to education and school fit neatly with the psychiatric literature on detribalisation and mental breakdown that appeared in East Africa from the 1930s.^76^ Missionary doctors were well aware of these theories, both from their reading of the *East African Medical Journal* and from their engagement with local branches of the British Medical Association (BMA).^77^ The association between education and neurasthenia in a small number of the African patients at Mengo highlights one of the ways in which race and social class might have intersected. Just as with their European patients, the doctors at Mengo paid attention to the social and physical environment of their African patients. The idea that Africans whose contact with civilisation had supposedly opened them up to mental illness might break down in different ways—culminating in either neurasthenia or hysteria—was just one of a myriad of assumptions made by the doctors. In this way, the breakdown of Africans who were unable to cope with a ‘civilised’ environment was not dissimilar to the breakdown of Europeans who were deemed to be physically and psychologically unprepared for the ‘tropical’ environment.

While the European patients may have understood some of the social and medical implications of neurasthenia, it is unclear how the African patients viewed the diagnosis. Certainly, if the doctors explained that neurasthenia would not ‘spoil the brain’ it would have gone some way towards relieving the anxiety of the Ganda patients.^78^ Conditions like neurasthenia had no direct equivalents in Luganda, the language of the dominant patient group at Mengo. Only *akawango* came close to the signs and symptoms of neurasthenia, being a ‘persistent headache on top of the head, usually lasting for months or years’ that due to its association with the brain frequently brought on depression and anxiety.^79^

Without the social uses of the diagnosis that were so important for validating the physical and mental complaints of many of the European patients, it is difficult to ascertain what purpose, if any, the diagnosis served for the African patients. A medical certificate could mean some relief from taxation or help secure a period of leave from work or school. It would be wrong, however, to dismiss their complaints outright, just as it would be to suggest that Africans were failing to cope with ‘civilisation’. Instead, these cases need to be seen as attempts of general medical practitioners to make sense of a wide range of vague and unidentifiable symptoms. As with the European patients, these attempts were not disconnected from ideas about race and class, but were above all shaped by the needs and expectations of the examination room.

## Conclusion

Barbara Sicherman has argued that neurasthenia ‘emphasized what physicians could do for their patients rather than their impotence’.^80^ In the context of a mission hospital, where options for specialist treatment were limited and the doctors were keen to influence minds and souls, this assessment certainly fits. Regardless of race, gender or class, the neurasthenic patients all received similar treatments, including bed rest, nutritious food, tonics, bromides, purgatives and blisters. These therapies had been popular in the treatment of ‘nervousness’ in Britain since the nineteenth century, and remained so at Mengo until the 1940s.^81^ While all patients at Mengo had to attend religious services on the wards, there is no evidence in the case notes to suggest that spiritual care made up a formal part of treatment. What is clear is that the doctors at Mengo had reason to believe they could treat neurasthenia successfully. Of the 98 cases of neurasthenia diagnosed in Europeans and Africans between 1906 and 1950, 24 were discharged ‘cured’, 51 ‘improved' and five ‘relieved’.

The ability of these doctors to state that they had identified the problem and could take steps to treat it must have had, in its own way, a positive psychological effect on the doctors. Faced with an overwhelming number of potential causes for each symptom, including malaria parasites, hookworm ova or some cause yet to be identified by tropical medicine, these doctors had more alternative diagnoses to rule out than their counterparts in America and Europe. Added to this was the general strain of working in a highly popular yet understaffed mission hospital. Such was the volume of correspondence, Albert Cook noted in 1919, coming from ‘all over both Protectorates, sometimes entailing 20 letters a day, all the Administrative work of a large Hospital, keeping in touch with Govt Officials [*sic*], looking after Affiliated Dispensaries, needful Indents, as well as the purely Professional side of the work’, that he felt exhausted and in desperate need of a secretary.^82^ It came as little surprise to those working in CMS mission headquarters the following year, when Cook started fainting during operations.^83^

In the busy, pressurised context of the hospital examination room, the diagnosis of neurasthenia was not just a way of policing white deviancy or an expression of whiteness under threat. The suffering of the patients was a clinical reality, and it is necessary to bear this in mind when considering neurasthenia, or indeed any medical diagnosis. Doctors were not alone in the examination room, and had to contend with the ideas and expectations of their patients as much as with their own assumptions. Here, the race of the patient was not the only consideration, and, through their search for somatic signs of ill-health, the doctors perhaps saw more similarities between their African and European patients than they did differences. For both groups of patients, the diagnosis was given to those patients who not only expressed a myriad of physical and mental complaints, but whose environment was such that the doctors felt that this diagnosis was appropriate. This included Europeans who had spent long periods of time in East Africa, but also educated and elite Africans.

Although the neurasthenia diagnosis died out in the 1940s, theories about detribalisation, education and mental breakdown continued to shape psychiatry in Africa until the 1960s, eventually feeding into Raymond Prince's ‘brain fag’ concept. This syndrome, which Prince applied to unmarried adult Nigerian males studying in England, was marked by ‘intellectual impairment, sensory impairment (chiefly visual), and somatic complaints most commonly of pain or burning in the head and neck’. These symptoms, Prince hypothesised, were ‘in some way related to the imposition of European learning techniques upon the Nigerian personality’.^84^

As a diagnosis, neurasthenia ultimately highlighted the cultural difficulties faced by both doctor and patient in the examination room, notably in the African patient's lack of the shared language of ‘nerves’. As such conditions became more visible for doctors in East Africa, concerns would be raised about the cultural gulf between European medical practitioners and African patients.^85^ If doctors were to be successful in treating minor psychological disease, they needed to be capable of understanding their patients. These concerns would, in turn, prompt significant developments in mental health care provision in Uganda and result in the training of the first generation of African psychiatrists.

## Notes

1 The idea of difference is a recurrent theme in the historical literature on psychiatry in Africa. See, for example, Deacon, ‘Madness, Race and Moral Treatment’; Jackson, *Surfacing Up*; McCulloch, *Colonial Psychiatry*; Vaughan, *Curing Their Ills*; Sadowsky, *Imperial Bedlam*.

2 Carothers, ‘Study of Mental Derangement’, 153–54.

3 Crozier, ‘What Was Tropical?’.

4 Beard, ‘Neurasthenia’.

5 This feature has led to comparisons between neurasthenia, chronic fatigue syndrome, and myalgic encephalomyelitis (‘ME’). Wessely, ‘Old Wine’.

6 Anderson, *Colonial Pathologies*; Crozier, ‘What Was Tropical?’; Kennedy, *Islands of White*; Mahone, ‘Psychology of the Tropics’.

7 Crozier, ‘What Was Tropical?’, 545.

8 On the importance of medical missions in Uganda, see especially Kuhanen, *Poverty*; Foster, *Early History*; Hansen, *Mission, Church and State*.

9 See, for example, Case No. 339, vol.1, 1923, Mengo Hospital Case Notes (hereafter MHCN), Albert Cook Memorial Library, Makerere University, Uganda (hereafter ACMM).

10 On psychiatry in colonial Uganda, see Mahone, ‘Psychiatry’; Pringle, ‘Psychiatry's “Golden Age”’.

11 A small number of cases of neurasthenia can be found in the early admissions registers of Toro Hospital, Fort Portal, as well as Mulago Hospital, Kampala.

12 Historians who have drawn on these archives include Daniel, ‘The Early History’; Hansen, *Mission, Church and State*; Summers, ‘Intimate Colonialism’; Tuck, ‘Syphilis’; Zeller, ‘The Establishment’.

13 Campbell, *Race and Empire*; Crozier, ‘What Was Tropical?’, 529.

14 Campbell, ‘The Making of “American”’.

15 Burr, ‘Neurasthenia’, 725.

16 This shift has been described more generally in Vaughan, *Curing Their Ills*; Mahone, ‘Psychology of the Tropics’.

17 King, ‘Tropical Neurasthenia’, 1518.

18 Loughran, ‘Hysteria and Neurasthenia’, 37.

19 For an extensive discussion on neurasthenia, see especially the essays in Gijswijt-Hofstra and Porter, eds, *Cultures of Neurasthenia*.

20 Havelock Charles, ‘Neurasthenia’, 24.

21 Stannus, ‘Tropical Neurasthenia’, 340.

22 Ibid., 343.

23 See, for example, Shelley and Watson, ‘An Investigation’.

24 See, for example, Gordon, ‘Mental Capacity’; Gordon, ‘An Enquiry’, 333; Sequeria, ‘The Brain’.

25 Mahone, ‘The Psychology of Rebellion’; Pringle, ‘Investigating “Mass Hysteria”’.

26 The Uganda Protectorate Annual Medical Reports contain details of those invalided home from Uganda each year.

27 Colonial Office, *Colonial Office List 1919*, 411.

28 Account of Receipts & Expenditure for the Year Ending December 31st 1908, CMS M/F 1, 2, University of Birmingham Special Collections (hereafter UoBSC); Case No. 173, vol. 1, 1908, MHCN, ACMM. During this year there was one other case of mental ill-health among the African in-patients—hysteria in a female patient. Case No. 759, vol. 5, 1908, MHCN, ACMM.

29 Missionaries Invalided Home Uganda + Elgon Missions, n.d., CMS M/M 7, UoBSC.

30 ‘Uganda Mission: Mengo’, *Mercy and Truth* no. 211, 200.

31 With 23 African patients admitted, hysteria was the second most common diagnosis of mental ill-health at Mengo during this time.

32 Crozier, ‘What Was Tropical?’, 532.

33 King, ‘Tropical Neurasthenia’, 518; Trewartha, ‘Recent Thought’.

34 Sicherman, ‘Uses of a Diagnosis’, 33.

35 Anderson, *Colonial Pathologies*, 147.

36 Crozier, ‘What Was Tropical?’.

37 Sicherman, ‘Uses of a Diagnosis’, 40-1.

38 Case No. 139, vol. 1 (Annie-Walker), 1930, MHCN, ACMM.

39 Case No. 55, vol. 1A (Annie-Walker), 1927, MHCN, ACMM.

40 Case No. 157, vol. 1A (Annie-Walker), 1921, MHCN, ACMM.

41 Case No. 26, vol. 6 (Annie-Walker), 1932–33, MHCN, ACMM.

42 Case No. 4, vol. 1B (Annie-Walker), 1935, MHCN, ACMM.

43 Crozier, ‘What Was Tropical?’, 537.

44 Thomson, ‘Neurasthenia in Britain’, 83. On the divide between neurological and psychological interpretations of neurasthenia, see especially Campbell, ‘Making of “American”’; Lutz, ‘Varieties of Medical Experience’.

45 Case No. 64, vol. 1A (Annie-Walker), 1932, MHCN, ACMM.

46 Case No. 6, vol. 1A (Annie-Walker), 1939, MHCN, ACMM.

47 Case No. 73, vol. 10 (Annie-Walker), 1936–38, MHCN, ACMM. An interesting debate on whether missionaries were susceptible to neurasthenia can be found in Stannus, ‘Tropical Neurasthenia’.

48 Anderson, *Colonial Pathologies*, 144.

49 See Endfield, ‘Through Marsh and Mountain’. The language of ‘nerves’ was eventually replaced in missionary recruitment with the language of the ‘neuroses’. The importance of ‘mental balance’ was stressed in the *Report of C.M.S. Commission*.

50 Medical certificate, 11 March 1929, enclosed in letter from Stephen Schnapp, Old East African Trading Company, Kampala, 12 March 1929, Mengo Hospital Correspondence (hereafter MHC), ACMM.

51 Such was the popularity of Albert Cook that, on his retirement in 1934, mission authorities feared that his withdrawal into private practice would necessitate the closure of Mengo Hospital altogether. Letter from Bishop of Uganda to the Rev. H. D. Hooper, London, 13 March 1934, CMS G3 A7/O 1934, 40, UoBSC.

52 Letter from J. P. M. to Albert Cook, 17 April 1914, MHC, ACMM.

53 Case No. 447, vol. 5, 1912, MHCN, ACMM. On the history of the ‘rest cure', see especially Marland, ‘“Uterine Mischief'”.

54 Case No. 59, vol. 1B (Annie-Walker), 1935, MHCN, ACMM.

55 On the importance of a diagnosis, see especially Gosling and Ray, ‘The Right to Be Sick’.

56 Crozier, ‘What was Tropical?’, 546. See also Anderson, *Colonial Pathologies*; Kennedy, *Islands of White*.

57 Letter from Edith C. to Dr. Cook, 12 Jan. 1914, MHC, ACMM; Letter from J. C. to Dr. Cook, 3 May 1914, MHC, ACMM; Case No. 176, vol. 1, 1914, MHCN, ACMM.

58 Thomson, ‘Neurasthenia in Britain’, 79.

59 See, for example, ‘Nature's Remedy', *Uganda Herald*, 13 March 1935, 11.

60 
*Health Instructions for Missionaries Overseas* (London, CMS, 1946), 3, CMS M/M9, UoBSC.

61 Case No. 62, vol. 1A (Annie-Walker), 1928, MHCN, ACMM.

62 Case No. 164, vol. 2 (Annie-Walker), 1920, MHCN, ACMM.

63 Orley, *Culture and Mental Illness*, 50.

64 German, ‘Aspects of Clinical Psychiatry’, 470.

65 Arthur Kleinman noted a similar tendency to highlight physical complaints among Chinese patients. Until recently, neurasthenia was a common diagnosis in East Asia. Kleinman, ‘Neurasthenia and Depression’.

66 Case No. 683, vol. 2, 1918, MHCN, ACMM.

67 Case No. 173, vol. 1, 1908, MHCN, ACMM.

68 Case No. 759, vol. 5, 1908, MHCN, ACMM.

69 Medical Mission Accounts and Statistic Returns, 1908–1918, CMS M/F 1, UoBSC.

70 Havelock Charles, ‘Neurasthenia’, 3. On mental ability and race, see especially Gould, *Mismeasure of Man*; Stepan, *The Idea of Race*.

71 There are no comments on the education or schooling of the female patients during the 1930s. While this might have reflected ideas of gender difference, it more likely reflects unequal opportunities for female education at that time. On the education of women in Uganda, see Kyomuhendo and McIntosh, *Women*; Nakawooya, ‘History of Muslim Women’.

72 Case No. 588, vol. 7, 1937, MHCN, ACMM.

73 On the role of King's College Budo in the development of political movements in Uganda, see especially Ssekamwa, *History and Development*; Summers, ‘Young Buganda’; Summers, ‘“Subterranean Evil”’.

74 Case No. 36, vol. 5, 1937, MHCN, ACMM.

75 Case No. 580a, vol. 6, 1939, MHCN, ACMM.

76 On this literature, see especially McCulloch, *Colonial Psychiatry*; Vaughan, *Curing Their Ills*.

77 See, for example, Davies, ‘The History’; ‘Meetings of Branches and Divisions’; Scott, ‘A Note’.

78 Orley, *Culture and Mental Illness*, 52–53.

79 Ibid., 11.

80 Sicherman, ‘Uses of a Diagnosis’, 39.

81 On the use of drugs in asylums and psychiatry in Europe, see Andrews *et al.*, *History of Bethlem*, 522; Berrios and Porter, eds, *History of Clinical Psychiatry*; Healy, *The Creation*; Shepherd and Wright, ‘Madness’.

82 Letter from Albert Cook to Dr Lankester, 18 Feb. 1919, CMS G3 A7/O 1919, 35, UoBSC.

83 Letter from Rowling to Manley, 5 Aug. 1920, CMS G3 A7/O 1920, 22, UoBSC.

84 Prince, ‘“Brain Fag”’, 559, 569.

85 See, for example, Trowell, ‘Training Medical Practitioners’.
